# α-Pheromone Precursor Protein Foc4-PP1 Is Essential for the Full Virulence of *Fusarium oxysporum* f. sp. *cubense* Tropical Race 4

**DOI:** 10.3390/jof9030365

**Published:** 2023-03-16

**Authors:** Lu Liu, Yinghua Huang, Handa Song, Mei Luo, Zhangyong Dong

**Affiliations:** 1 Innovative Institute for Plant Health/Key Laboratory of Green Prevention and Control on Fruits and Vegetables in South China, Ministry of Agriculture and Rural Affairs, Zhongkai University of Agriculture and Engineering, Guangzhou 510225, China; 2 Guangdong Agribusiness Tropical Crop Science Institute, Maoming 525100, China

**Keywords:** *Fusarium oxysporum* f. sp. *cubense*, virulence, α-pheromone precursor

## Abstract

*Fusarium oxysporum* f. sp. *cubense* (*Foc*), which causes Fusarium wilt of bananas, is considered one of the most destructive fungal pathogens of banana crops worldwide. During infection, *Foc* secretes many different proteins which promote its colonization of plant tissues. Although *F. oxysporum* has no sexual cycle, it has been reported to secrete an α-pheromone, which acts as a growth regulator, chemoattractant, and quorum-sensing signaling molecule; and to encode a putative protein with the hallmarks of fungal α-pheromone precursors. In this study, we identified an ortholog of the α-pheromone precursor gene, *Foc4-PP1*, in *Foc* tropical race 4 (TR4), and showed that it was necessary for the growth and virulence of *Foc* TR4. *Foc4-PP1* deletion from the *Foc* TR4 genome resulted in decreased fungal growth, increased sensitivity to oxidative stress and cell-wall-damaging agents, and attenuation of pathogen virulence towards banana plantlets. Subcellular localization analysis revealed that Foc4-PP1 was concentrated in the nuclei and cytoplasm of *Nicotiana benthamiana* cells, where it could suppress BAX-induced programmed cell death. In conclusion, these findings suggest that Foc4-PP1 contributes to *Foc* TR4 virulence by promoting hyphal growth and abiotic stress resistance and inhibiting the immune defense responses of host plants.

## 1. Introduction

Fusarium wilt of banana (also named Panama disease), which is caused by the soilborne fungus *Fusarium oxysporum* f. sp. *cubense* (*Foc*), is a devastating disease which results in severe economic losses [1]. *Foc* is divided into four physiological races based on susceptible banana cultivars [2]: race 1 attacks bananas of the genome groups AAB and ABB and was responsible for the Gros Michel epidemics in the 1950s. Race 2 infects only cooking bananas of cv. Bluggoe (ABB. Race 3 is no longer considered a *Foc* race because these isolates do not cause disease in banana but rather in *Heliconia* spp. [3,4]. Race 4 infects all the hosts of races 1 and 2 and the Cavendish cultivars (AAA) and is currently regarded as the most virulent Fusarium wilt pathogen [5]. According to geographical characteristics and temperature adaptation profiles, *Foc* race 4 is further divided into tropical and subtropical race 4 (TR4 and STR4, respectively) [1]. *Foc* TR4 is considered the most destructive *Foc* variety, as it attacks almost all banana species and has more transmission routes and stronger pathogenicity compared to the other variants [6,7,8]. There are still no effective strategies for controlling *Foc* TR4 because of its stress resistance [9] and because our understanding of the molecular mechanism underlying *Foc* TR4 pathogenicity is currently very limited.

In the process of invasion and colonization of banana plants, *Foc* secretes a series of factors that promote its infection of the host, including cell-wall-degrading enzymes [10], toxins [11], and small proteins called effectors which help evade the plant’s immune system [12]. For example, FocSix1 and FocSix8—homologs of secreted in xylem (SIX) effectors originally identified in the xylem sap proteome of tomato plantlets inoculated with *F. oxysporum* f. sp. *lycopersici* (*Fol*)—are necessary for the full virulence of *Foc* [13,14]. It has been shown that a cerato-platanin protein FocCP1 and metalloprotease FocM35_1 are important *Foc* TR4 virulence factors which promote pathogen penetration and inhibit host immunity, respectively, and are required for *Foc* TR4 full virulence [15,16]. However, to date, only a few *Foc* effectors have been characterized. Therefore, it is necessary to identify others *Foc* effectors to better understand the molecular mechanisms underlying the pathogenic colonization of banana plants.

In a model organism *Saccharomyces cerevisiae*, haploid cells of opposite mating types (MATa and MATα) secrete pheromones a-factor lipopeptide and α-factor peptide, respectively, which can bind only mating-type-specific cell surface receptors, Ste3p and Ste2p, respectively [17,18,19,20]. Binding of α-pheromone to Ste2p activates the pheromone-response-related signaling pathway of mitogen-activated protein kinase (MAPK) consisting of Ste11p, Ste7p, and Fus1p, which leads to cell cycle arrest, oriented growth towards neighboring cell/plasma membranes, and nuclear fusion of the two mating partners [21,22]. *MFα1* and *MFα2* genes encode α-factor precursors which contain four and two repeats of 13 residue mature α-factors, respectively [23]. On its N- and C-terminal borders, each mature peptide repeat is flanked by cleavage signals for Ste13p and Kex2p peptidases, respectively [24], which are thought to be a characteristic of α-pheromone precursors [25]. Generally, all the copies of the mature α-factor are cleaved and released by the cell [26].

In this study, we characterized Foc4-PP1, an ortholog of the α-pheromone precursor, which is expressed in *Foc* TR4. Our results revealed that Foc4-PP1 contributed to the complete virulence of *Foc* TR4, indicating that Foc4-PP1 is a potential virulence factor for this pathogen. We also demonstrated that ΔFoc4-PP1 deletion mutants had higher sensitivity to oxidative stress and cell-wall-damaging agents and a slower growth rate than the wild type (WT) strain. Foc4-PP1 suppressed BAX-induced cell death in *Nicotiana benthamiana* and was localized in the nuclei and cytoplasm of plant cells. Our results indicate that Foc4-PP1 plays an essential role in the growth, development, resistance to oxidative stress, and virulence of *Foc* TR4.

## 2. Materials and Methods

### 2.1. Plant Material and Fungal Strains

Banana plantlets (Musa AAA Cavendish subgroup, cv. Brazil) with five to six leaves were cultivated in a greenhouse at 27 °C under a light regime of 12:12 h (light:dark). *N. benthamiana* was grown in a greenhouse at 25 °C under the same light regime. *Foc* TR4 14013 was used as the WT strain in this study. All *Foc* TR4 strains were grown and subcultured on potato dextrose agar (PDA). For experiments with banana inoculation, fungi were grown in potato dextrose broth (PDB) at 28 °C with agitation at 180 rpm for 5–10 days.

### 2.2. CDS Amplification and Sequence Analysis

Total RNA was isolated from fresh hyphae cultured for around 6 d on PDA plates using the RNA Simple Total RNA Kit (TianGen Biotech, Beijing, China). cDNA was synthesized using the TransScript One-Step gDNA Removal and cDNA Synthesis SuperMix kit (Transgen Biotech, Beijing, China). The *Foc4-PP1* full-length cDNA sequence was amplified using Foc4-PP1F and Foc4-PP1R primers. All primers were synthesized by Tianyi Huiyuan Biotech (Guangzhou, China); their full sequences are presented in Table S1.

The signal peptide in Foc4-PP1 was predicted using SignalP-5.0. Protein domains or motifs were predicted by NCBI CDD analysis “http://www.ncbi.nlm.nih.gov/Structure/cdd/wrpsb.cgi” (accessed on 2 January 2023). Foc4-PP1 homologous proteins were identified by searching non-redundant protein sequence database (NRDB) using the BLASTP program at NCBI “http://www.ncbi.nlm.nih.gov” (accessed on 2 January 2023). Homologous proteins of Foc4-PP1from other fungi were selected and further compared using ClustalW. PSORT II “https://www.genscript.com/psort.html” (accessed on 2 January 2023), TMHMM 2.0 “https://services.healthtech.dtu.dk/service.php?TMHMM-2.0” (accessed on 2 January 2023), and GPI-SOM “http://gpi.unibe.ch/” (accessed on 2 January 2023) were used to predict subcellular localization, transmembrane domains, and glycosylphosphatidylinositol (GPI) anchoring sites, respectively.

### 2.3. Generation of Foc4-PP1 Deletion Mutants

*Foc4-PP1* targeted deletion mutants were constructed as previously reported [27]. Briefly, 1000 bp sequences of the 5′- and 3′-flanking regions in the *Foc4-PP1* gene were amplified using two pairs of primers, Foc4-PP1-AF/AR and Foc4-PP1-BF/BR (Table S1), Phanta Max polymerase (Vazyme, Nanjing, China), and the genomic DNA of strain 14013 as a template. A 1692 bp DNA fragment of the *Foc4-PP1* gene was replaced with the hygromycin B resistance (*HygR*) and green fluorescent protein (*GFP*) cassette driven by a constitutive TrpC promoter amplified from the pCT74 vector. In this study, we used polyethylene glycol-mediated protoplast transformation. Two-hundred microliter protoplasts of *Foc* TR4 14013 were mixed with 3–5 μg linearized vectors and incubated on ice for 20 min. Then, we added 1 mL of 40% PEG to the mixture and inverted gently. After standing for 10 min, STC buffer (1 M sorbitol, 50 mM Tris-Cl, 10 mM CaCl_2_, pH 7.4) was added, and the mixture was inverted gently. Then, 3 mL liquid regeneration medium (1 g/L yeast paste, 1 g/L casein, 6 M sucrose) was added into the protoplasts, which were then cultured overnight at 28 °C, 100 rpm. The regenerated protoplasts were mixed with a regeneration medium with 1% agar (containing 50 μg/mL hygromycin B) at about 50 °C. They spread out on a Petri dish at 25 °C for 2–3 d for mutant selection. Putative deletion mutants were identified by PCR using primer pairs Foc4-PP1-F1/F2, F3/F4, and F5/F6 (Table S1) and further confirmed by sequencing (Tianyi).

### 2.4. Pathogenicity Assays

Banana plantlets (Musa sp. AAA cv. Brazilian) at the stage of five–six leaves were selected for pathogenicity testing by the method described previously [28]. After culturing on PDA for 7 days, the conidia of the WT and Δfoc4-PP1 mutant strains were collected, washed and resuspended in sterile distilled deionized water to a final concentration of 10^6^ conidia per mL, and 50 mL of the conidial suspension was used to inoculate banana plantlets by irrigation of the roots. Disease symptoms in the banana pseudostem were recorded at 28 days post inoculation. The disease was scored as follows: 0 (no symptoms), 1 (some brown spots on the inner rhizome), 2 (browning of less than 1/4 of the inner rhizome), 3 (browning of up to 3/4 of the inner rhizome), and 4 (dark brown color and death of the entire inner rhizome and pseudostem) [29]. Three replicates per treatment (10 plantlets per replicate) were used to calculate the disease scores. The data were analyzed using ANOVA in SPSS. The disease index (DI) was calculated using the following formula:

DI = (100 × ∑ (n × s))/(N × 5),

where “n” is the number of banana plants with the corresponding disease score (s) and “N” is the total number of banana plants tested.

### 2.5. Growth, Stress Sensitivity, and Cellophane Membrane Assays

To determine phenotypic differences between the WT and ΔFoc4-PP1 strains, 5 mm mycelial plugs of each strain were inoculated on the PDA plates for 6 days, and the colony diameters and growth rates were recorded and analyzed with Duncan’s multiple range tests. To determine the differences in stress responses, the WT and mutant strains were cultured on PDA plates supplemented with 1 M NaCl, 1 M sorbitol, or 25 mM hydrogen peroxide (H_2_O_2_) for 6 days at 28 °C. The sensitivity to cell-wall-disrupting agents was tested on PDA supplemented with Congo red (200 µg/mL). The penetration ability of each strain was tested on a cellophane membrane as previously described [15]. The experiments were repeated thrice. All data were analyzed by SPSS. The inhibition ratio was calculated using the following formula:

Inhibition ratio = (Diameter of control sample − Diameter of treated sample)/Diameter of control sample × 100%

### 2.6. Subcellular Localization and Cell Death Suppression Assay

To determine the subcellular localization of the Foc4-PP1 protein, the coding sequence of *Foc4-PP1* with or without the signal peptide was fused with the *eGFP* gene [30] and inserted into pCambia 1305.1 to generate pCambia 1305:Foc4-PP1:eGFP and pCambia 1305:Foc4-PP1^∆sp^:eGFP constructs, respectively. The resulting plasmids were used to transform *Agrobacterium tumefaciens* strain GV3101 suspended at OD_600_ = 0.8 in the infiltration medium (10 mM MES, pH 5.5, and 200 μM acetosyringone), and *Agrobacterium* cells carrying the constructs were applied to *N. benthamiana* leaves. The vehicle plasmid containing *eGFP* alone was used as a control. At 48 h post infiltration, leaves were examined under a Nikon ECLIPSE Ni microscope (Nikon, Tokyo, Japan).

In the next experiment, the Foc4-PP1^∆sp^ coding sequence was cloned into pCambia 1305.1 to generate pCambia 1305:Foc4-PP1^∆sp^. pCambia 1305:BAX and pCambia 1305:eGFP were used as positive and negative controls, respectively. The constructs were used to transform *A. tumefaciens* GV3101, which was then infiltrated into *N. benthamiana* leaves. After 24 h, the same infiltration sites were injected with *Agrobacterium* cells carrying pCambia 1305:BAX. Photographs for phenotype analysis were taken 5 d after the last infiltration. The experiment was performed in triplicate.

## 3. Results

### 3.1. Sequence Analysis of the Foc4 PP1 Gene and Protein

**Figure 1:**
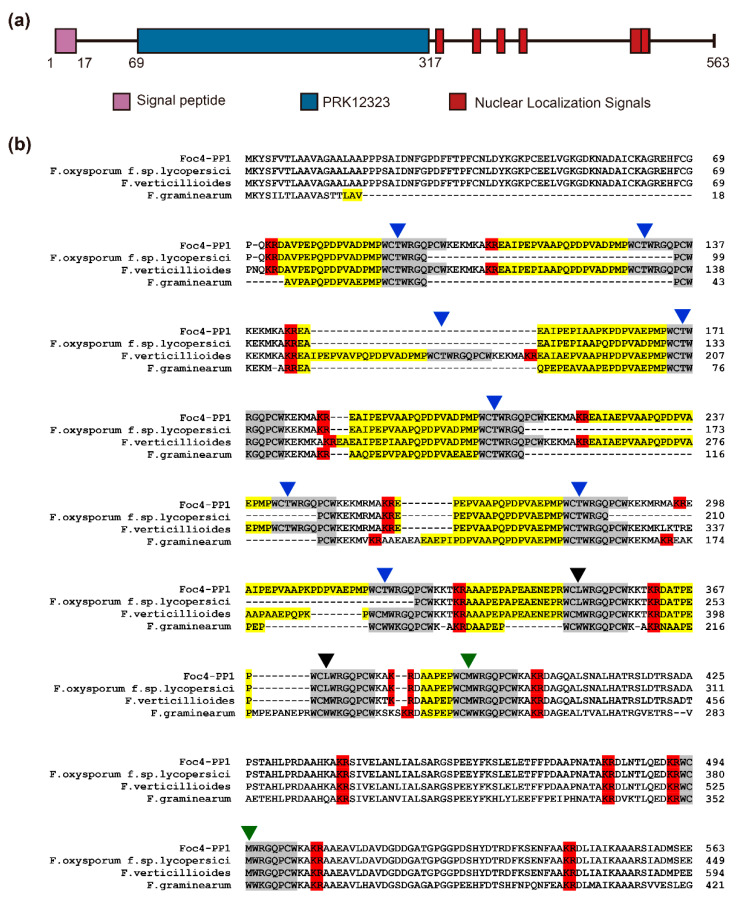
Sequence analysis of the Foc4-PP1. (**a**) The domain prediction of Foc4-PP1. The six putative nuclear localization signals are ^325^PCWKKTK^331^, ^355^PCWKKTK^361^, ^376^PCWKAKR^382^, ^396^PCWKAKR^402^, ^500^PCWKAKR^506^, and ^491^KRWCMWRGQPCWKAKRA^507^, respectively. (**b**) Multiple sequence alignment of the deduced Foc4-PP1 amino acid sequences with predicted α-pheromone precursors from *F. oxysporum* f. sp. Lycopersici (FOXG_08636), *F. graminearum* (FGSG_05061), and *F. verticillioides* (FVEG_06038). Predicted KR and RR cleavage signals for KEX2-like endopeptidases are highlighted in red. Predicted maturation signals characterized by the presence of XA or XP dipeptide repeats are highlighted in yellow. Predicted mature α-pheromone decapeptide repeats are highlighted in grey. Colored arrowheads indicate differences between the decapeptide repeats at the third amino acid.

The *Foc4-PP1* gene was first identified by RNA-seq profiling of banana roots infected with *Foc* TR4. The *PP1* sequence (GenBank accession No. OQ425405) in *Foc* TR4 contains 1692 bp and encodes a protein of 563 amino acids with an N-terminal signal peptide (cleavage site between residues 17 and 18) (Figure 1a) but without a transmembrane structure or GPI-anchoring site. In addition, Foc4-PP1 contains six putative nuclear localization signals: ^325^PCWKKTK^331^, ^355^PCWKKTK^361^, ^376^PCWKAKR^382^, ^396^PCWKAKR^402^, ^500^PCWKAKR^506^, and ^491^KRWCMWRGQPCWKAKRA^507^ (Figure 1a). PSORT II prediction analysis indicated that Foc4 PP1 should have nuclear and cytoplasmic localization (Table S2).

BLASTP search of the NCBI database revealed that Foc4-PP1 had a high degree of homology with pheromone precursor proteins from *Fusarium* spp., including those of *Fusarium* sp. *NRRL 52700* (GenBank accession no. KAF5624885.1, 96.98%), *F. oxysporum* f. sp. *albedinis* (KAI3574876.1, 92.33%), *F. nygamai* (AEP96279.1, 95.94%), *F. thapsinum* (AEP96281.1, 90.12%), and *F. thapsinum* (AEP96282.1, 89.61%). NCBI-CDD analysis showed that these *Fusarium* orthologs of pheromone precursor proteins, including Foc4-PP1, belong to the PRK12323 conserved protein domain superfamily. Multiple sequence alignment revealed that these pheromone precursor orthologs have the characteristics of fungal α-pheromone precursors observed in *Fusarium* [25,31], containing 11 α-pheromone decapeptide repeats with near-identical sequences and multiple Kex2p protease-like cleavage signals (usually KR or RR) (Figure 1b). These results suggest that *Foc4-PP1* is an α-pheromone precursor gene in *Foc* TR4 14013 and encodes an α-pheromone.

### 3.2. Deletion of Foc4-PP1 Compromises Fungal Growth and the Virulence of Foc TR4 14013 in Cavendish Banana

To characterize the functions of Foc4-PP1 in *Foc* TR4, we constructed a gene replacement fragment containing the *HygR* and *GFP* gene cassette (Figure S1a) and used it to knockout the *Foc4-PP1* region in the *Foc* TR4 14013 strain. Two *Foc4-PP1* deletion mutants (ΔFoc4-PP1-12 and ΔFoc4-PP1-13) were obtained (Figure S1b) and confirmed by PCR and sequencing.

**Figure 2:**
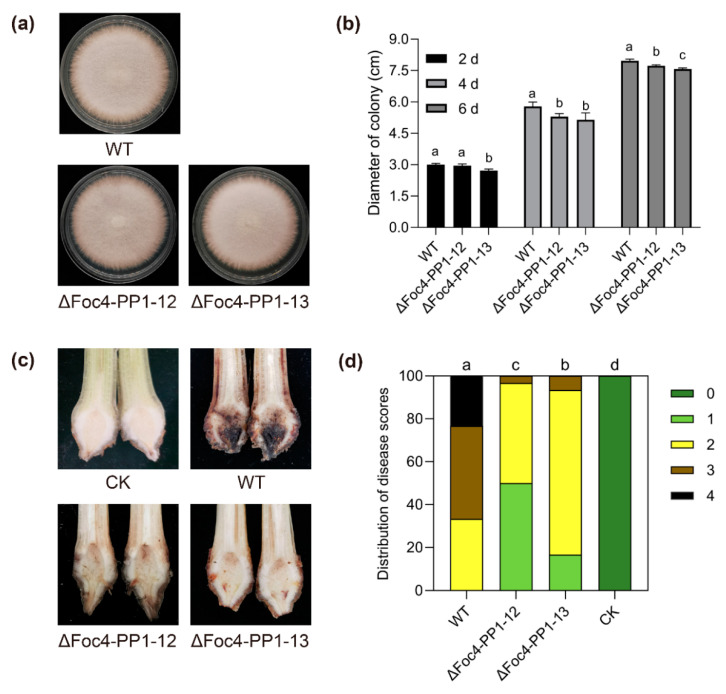
Foc4-PP1 contributes to the virulence of *Foc* TR4. (**a**) The colony morphologies of WT, ∆Foc4-PP1-12, and ∆Foc4-PP1-13 strains after growth on potato dextrose agar (PDA) plates for 6 d. (**b**) Colony diameters after growth for 2, 4, and 6 days. (**c**) Disease phenotype and (**d**) disease index distribution in banana plantlets inoculated with the WT, ∆Foc4-PP1-12, and ∆Foc4-PP1-13 strains at 28 days post inoculation. CK: banana plantlets inoculated with water; 0–4: different range of disease scores. Data presented in (**b**,**d**) are means ± SDs from three independent experiments. The values were analyzed with Duncan’s multiple range tests by SPSS. Columns with different letters indicate a significant difference (*p* < 0.05).

Phenotypic analysis indicated that ΔFoc4-PP1-12 and ΔFoc4-PP1-13 had slightly reduced colony growth rates compared with those of the WT (Figure 2a). Four days after inoculation, the colony diameters of ΔFoc4-PP1-12 and ΔFoc4-PP1-13 on PDA were less than those of the WT strain by 8.6% and 10.3%, respectively (Figure 2b). To investigate whether Foc4-PP1 is required for *Foc* TR4′s virulence, we inoculated Cavendish banana roots with the WT and Foc4-PP1 deletion mutants and observed the development of disease symptoms. At 28 days post inoculation, the symptoms in rhizome tissues were conspicuous enough to evaluate disease severity. The results showed that the virulence of ΔFoc4-PP1-12 and ΔFoc4-PP1-13 for banana plants was significantly reduced compared with that of the WT, as evidenced by decreased browning of rhizome tissues (Figure 2c) and lower disease severity indexes (Figure 2d). Among the plantlets inoculated with WT, for about 66.7% of them, the rhizome disease was score above three. In the plantlets treated with the two knock-out mutants of ΔFoc4-PP1-12 and ΔFoc4-PP1-13, only 3.3% and 6.7% of the rhizome showed a disease score above three, respectively; and plantlets inoculated with water (CK) showed no symptoms. These observations suggest that Foc4-PP1 is essential for the growth and full virulence of *Foc* TR4.

### 3.3. Sensitivity of the Foc4-PP1 Deletion Mutants to Abiotic Stresses

**Figure 3:**
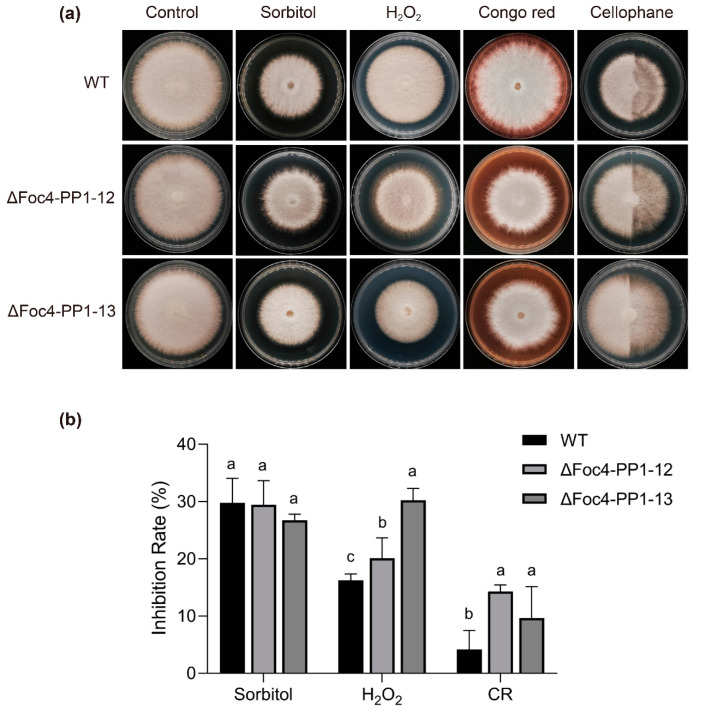
Sensitivity test of the WT and ΔFoc4-PP1 strains against abiotic stress. (**a**) Colony appearance of the WT, ∆Foc4-PP1-12, and ∆Foc4-PP1-13 strains on PDA plates supplemented with 1 M sorbitol, 25 mM H_2_O_2_, 200 μg/mL Congo red, and cellophane, respectively. Plates were incubated at 28 °C for six days after inoculation. (**b**) Quantification of colony size inhibition as appeared in (**a**). The strains cultured under non-stress conditions were used as controls. The inhibition ratio was calculated as the percentage growth reduction of the treated samples compared to the control. The values were analyzed with Duncan’s multiple range tests by SPSS. Columns with different letters indicate a significant difference (*p* < 0.05).

To explore the mechanisms underlying the role of Foc4-PP1 in *Foc* TR4 virulence, we assessed the sensitivity of the ΔFoc4-PP1 mutant strains to osmotic and oxidative stresses and cell-wall-damaging agents. ΔFoc4-PP1-12 and ΔFoc4-PP1-13 cultured on PDA supplemented with 25 mM H_2_O_2_ significantly inhibited fungal growth, reducing the colony diameter by 8.2% and 20.8%, respectively, relative to the WT (Figure 3a). The two mutant strains of ΔFoc4-PP1 were also more sensitive to a cell-wall-disrupting agent than the WT strain (Figure 3a). In detail, 200 μg/mL Congo Red inhibited the growth of ΔFoc4-PP1-12 and ΔFoc4-PP1-13 by 14.3% and 9.6%, respectively, and that of the WT strain only by 4.1%, indicating higher sensitivity of the mutants to cell-wall stress (Figure 3b). These results reveal the role of Foc4-PP1 in the susceptibility of *Foc* TR4 to oxidative and cell-wall-damaging agents. As tolerance of pathogenic fungi to oxidative and cell-wall stresses has been shown to be associated with their virulence [32,33], these findings suggest that an increase in *Foc* TR4 stress sensitivity due to *Foc4-PP1* deletion may account for the loss in virulence.

However, the two ΔFoc4-PP1 mutants differed in their responses to osmotic stress. In the culture with 1 M sorbitol, the growth trends of ΔFoc4-PP1-12 and ΔFoc4-PP1-13 were similar to those of WT. These data suggest that the ΔFoc4-PP1-12 and ΔFoc4-PP1-13 deletion mutants are less sensitive to osmotic stress caused by sorbitol than to oxidative stress and cell-wall damage.

In addition, we assessed the ability of the two ΔFoc4-PP1 mutants to penetrate a cellophane membrane. The growth of fungi on cellophane-covered plates can be used to simulate the penetration phase in vitro [34]. The ability of the ΔFoc4-PP1 mutants to penetrate cellophane was no different than that of the WT strain (Figure 3a), further confirming the notion that deletion did not reduce the host virulence of *Foc* TR4 towards banana by decreasing the penetration ability of mycelium.

### 3.4. Foc4-PP1-Mediated Suppression of BAX-Induced Cell Death in N. benthamiana

**Figure 4:**
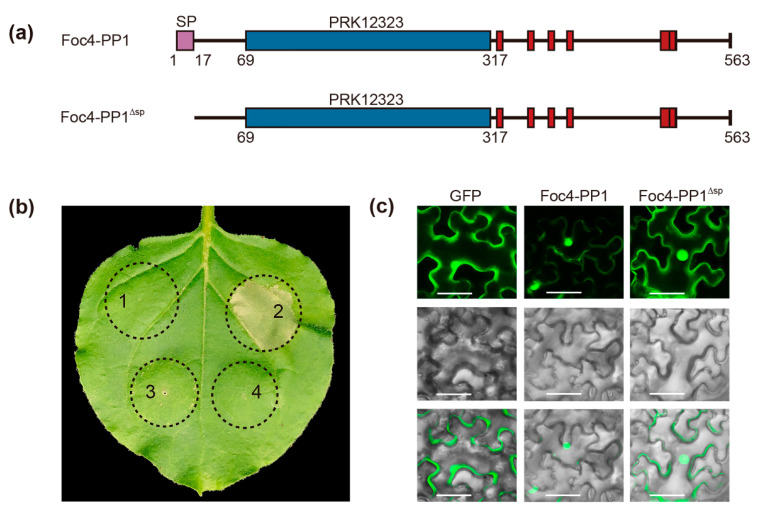
Foc4-PP1 suppresses BAX-induced cell death in *N. benthamiana*. (**a**) Schematic illustration of Foc4-PP1 without signal-peptide mutants used in this study. (**b**) Suppression of BAX-triggered cell death in *N. benthamiana* by Foc4-PP1. Infiltration with *Agrobacterium* strain GV3101 carrying pCambia 1305:Foc4-PP1, pCambia 1305:BAX, pCambia 1305:Foc4-PP1^∆sp^, and pCambia 1305:GFP. 1: pCambia 1305:Foc4-PP1; 2: pCambia 1305:BAX; 3: pCambia 1305:Foc4-PP1^∆sp^; 4: pCambia 1305. *N. benthamiana* leaves were further challenged with *Agrobacterium* cells carrying BAX at the same sites. The cell death phenotype was scored and photographs were taken 5 days after the last infiltration. (**c**) Subcellular localization of Foc4-PP1:eGFP and Foc4-PP1^∆sp^:eGFP were observed by Nikon ECLIPSE Ni microscope at 48 h after *Agrobacterium*-mediated transformation of *N. benthamiana* and merged GFP, and bright-field images are shown. The vector was used as a control. Bars = 50 μm. eGFP, enhanced green fluorescent protein.

To investigate the influence of Foc4-PP1 on plant immunity, Foc4-PP1 and its signal peptide-deficient mutant (Foc4-PP1^∆sp^) were transiently expressed in *N. benthamiana* (Figure 4a). The expression of GFP and of a mammalian proapoptotic factor BAX were used as negative and positive controls. Twelve hours after the first inoculation, BAX-expressing *Agrobacterium* was applied at the same sites. The Bax protein is a member of the B-cell lymphoma-2 (BCL-2) family, which control the intrinsic apoptosis pathway [35]. The BAX-triggered cell death symptoms in *N. benthamiana* resembled the hypersensitive response by the plant immune system [36]. We found that Foc4-PP1 and Foc4-PP1^∆sp^ did not induce cell death. Moreover, cell death triggered by BAX in the leaves co-expressing BAX and Foc4-PP1^∆sp^ was almost completely suppressed (Figure 4b). These results indicate that Foc4-PP1^∆sp^ could inhibit BAX-induced cell death, suggesting its role in the modulation of plant immunity.

To analyze subcellular localization of Foc4-PP1 in plants, Foc4-PP1:eGFP and Foc4-PP1^∆sp^:eGFP were transiently expressed in *N. benthamiana*. Green fluorescence of Foc4-PP1:eGFP and Foc4-PP1^∆sp^:eGFP was detected both in the nucleus and cytoplasm; similar results were obtained for the control (GFP) (Figure 4c).

## 4. Discussion

An increasing number of effectors have been reported in fungal pathogens of plants, but only a few of them have been experimentally characterized in *Foc* TR4. In our previous study, we have predicted a series of effector-coding genes using the well-annotated genome of *Foc* TR4 14013. In this study, we identified and characterized the *Foc4-PP1* gene, which encodes an α-pheromone precursor protein of 563 amino acids. Our findings indicate that Foc4-PP1 regulates the growth of *Foc* TR4 and is required for its full virulence in banana plants. The deletion of *Foc4-PP1* resulted in a certain reduction in fungal tolerance to oxidative stress and a cell-wall-damaging agent. Furthermore, Foc4-PP1^∆sp^ suppressed BAX-induced cell death in *N. benthamiana.* Subcellular localization analysis revealed Foc4-PP1 both in the nuclei and in the cytoplasm of plant cells.

Although orthologous α-pheromone-related genes have been described in numerous yeasts and filamentous ascomycetes, they differ in their functional activity. In heterothallic ascomycetes, α-pheromones are essential for successful mating and are produced in a mating-type-specific manner [37,38]. In homothallic and asexual ascomycetes, α-pheromone-related genes often perform additional functions not associated with sexual reproduction. For example, in *Candida albicans* α-pheromones are necessary for intercellular signaling during biofilm formation [39]. α-pheromones are dispensable for self-fertility in a homothallic ascomycete *Gibberella zeae* [40] but can induce chemotaxis of growing hyphae in *Fusarium graminearum* [41]. An α-pheromone secreted by asexual *F. oxysporum* elicits chemotropic reactions in germ tubes [31] and inhibits fungal growth and cell division in a Ste2-independent manner [42]. The interaction of α-pheromones with the plasma membrane G-protein-coupled receptor Ste2 leads to the activation of the MAPK cell-wall integrity (CWI) cascade to control conidial germination in a density-dependent manner [43]. In this study, we found that the knockout of the α-pheromone precursor gene *Foc-PP1* in *Foc* TR4 decreased the pathogen virulence for the banana host, suggesting that α-pheromones may play a previously undiscovered role in fungal virulence, thereby broadening the functional activity spectrum of α-pheromones in asexual fungi.

Although the mechanisms underlying the role of Foc-PP1 in fungal virulence are not yet defined, Foc-PP1 seems not to reduce the host virulence of *Foc* TR4 by decreasing the penetration ability of mycelium. We speculate the mechanisms underlying the role of Foc-PP1 in fungal virulence may be related to the regulation of mycelial growth and cell-wall integrity because the *Foc4-PP1* knockout mutants exhibited a slower growth rate and higher sensitivity to cell-wall stress compared to the WT. These results point to the involvement of α-pheromone not only in the chemotactic growth of mycelia via the CWI pathway [31], but also in the maintenance of cell-wall integrity in *F. oxysporum*. Pheromone-induced degradation and fusion of cell walls between cells have been extensively reported in fungi, as both mating and polarized growth are accompanied by locally remodeling of the cell wall [44,45,46]. Especially during mating, cells must degrade the intervening cell wall to allow fusion of the partners. Pheromone-induced degradation and fusion of intercellular cell walls in *saccharomyces cerevisiae* was accomplished through the mating signaling pathway, which involves the detection of extracellular α-pheromone by Ste2 receptors, activation of MAPK cascades, and coordination of downstream intracellular signal transduction pathways, and have been found to be highly conserved in fungal genomes [47,48]. It is noticeable that in the asexual pathogen of *F. oxysporum*, which causes wilt diseases in crops, α-pheromone is an autocrine signal. The autocrine signal α-pheromone controls the conidial germination via the CWI MAPK cascade, which is distinct from the chemotactic cascade in *Saccharomyces cerevisiae* [43]. Meanwhile, the physiological role of α-pheromone in controlling conidial germination also provides more possibilities for its role in host infection in *F. oxysporum*, as the spore germination is an important event for *F. oxysporum* initial invasion.

Foc4-PP1 inhibited programmed cell death triggered by the pro-apoptotic protein BAX in *N. benthamiana*, suggesting that its contribution to fungal virulence includes suppression of defense-related host-cell death. In contrast to the α-pheromone of *Saccharomyces cerevisiae*, its ortholog in *F. oxysporum* contains two cysteines, which are thought to have a function in redox-regulated processes [42,49]. This idea seems to be consistent with our finding that the Foc4-PP1 knockout mutants have higher sensitivity to H_2_O_2_-induced oxidative stress, suggesting that the α-pheromone may indeed play a yet unknown role in redox-regulated events in *F. oxysporum*. Interestingly, the two-cysteine oxidation version of α-pheromone eliminates its chemoattractant activity [49]. *F. oxysporum* shared the ste2 receptor for chemotaxis to α-pheromone and plant peroxidases (Prx) secreted by tomato roots [31]. The deletion of ste2 resulted in the loss of chemotaxis to α-pheromones and Prx [50].

In conclusion, we identified a virulence factor, the α-pheromone precursor gene *Foc4-PP1*, which promotes *Foc* TR4 infection of banana plants. The deletion of *Foc4-PP1* slowed fungal growth and increased sensitivity to oxidative and cell-wall stresses. Our results reveal a novel function of α-pheromones, which broadens understanding of the role played by these effectors in asexual fungi and opens new research directions. Future studies should focus on the disclosure of molecular mechanisms underlying the contribution of Foc4-PP1 to the virulence of *Foc* TR4 fungi for bananas.

## Data Availability

Not applicable.

## Supplementary Materials

The following supporting information can be downloaded at: https://www.mdpi.com/article/10.3390/jof9030365/s1. Figure S1: A schematic representation of targeted deletion of the Foc4-PP1 gene. Table S1: Oligonucleotide primers used in this study. Table S2: Prediction of the subcellular localization of Foc4-PP1.
