# Supplementary material for: α-Pheromone Precursor Protein Foc4-PP1 Is Essential for the Full Virulence of Fusarium oxysporum f. sp. cubense Tropical Race 4
Source: J Fungi (Basel). 2023 Mar 16;9(3):365. doi: 10.3390/jof9030365 (PMC10057649; doi:10.3390/jof9030365)
Supplement: Supplementary file 1 [file jof-09-00365-s001.zip › jof-2261639-supplementary.pdf]

Journal of Fungi

Table S1. Oligonucleotide primers used in this study.

Primer	Sequence（5′-3′）	Tm / ℃	Amplicon
Foc4-PP1-AF	TCCggtaccAAAGCATTGGCCCCACTATC	60	993 bp
Foc4-PP1-AR	TGGctcgagATCGAGCATCTTCTAACATCTTCAGG		
Foc4-PP1-BF	TCCgaattcTTCGATGGGAACGAGTCTGA	59	817 bp
Foc4-PP1-BR	TGGtctagaAACTGCCGATGAACTTGTGA		
Foc4-PP1-F1	GAAGCCCTTCAACTTCTCTCGT	60	1279 bp
Foc4-PP1-F2	TCTATCAGAGCTTGGTTGACG		
Foc4-PP1-F3	CTACGAGCTGCTGCCTTGAT	58	1464 bp
Foc4-PP1-F4	CGGTCCTCAGAAGCGTGATG		
Foc4-PP1-F5	CGGTCTTGCGATGATTATCA	60	1134 bp
Foc4-PP1-F6	CAAGTACCTGCGTCGTCAG		
Foc4-PP1F	CACCAAATCGACtctagaATGAAATACTCCTTCGTTAC	60	1692 bp
Foc4-PP1R	TCACCATGGTGGCggtaccTTCCTCGGACATGTCGGC		
Foc4-PP1-SPF	CACCAAATCGACtctagaATGGCGCCTCCGCCGTCC	60	1641 bp
Foc4-PP1-SPR	TCACCATGGTGGCggtaccTTCCTCGGACATGTCGGC		

Recognition sequences for restriction enzymes are underlined.

Table S2. Prediction of subcellular localization of Foc4-PP1.

Protein	PSORTII prediction
Foc4-PP1	Nuclear, 82.6%<br>Cytoplasmic, 13.0%<br>Cytoskeletal, 4.3%

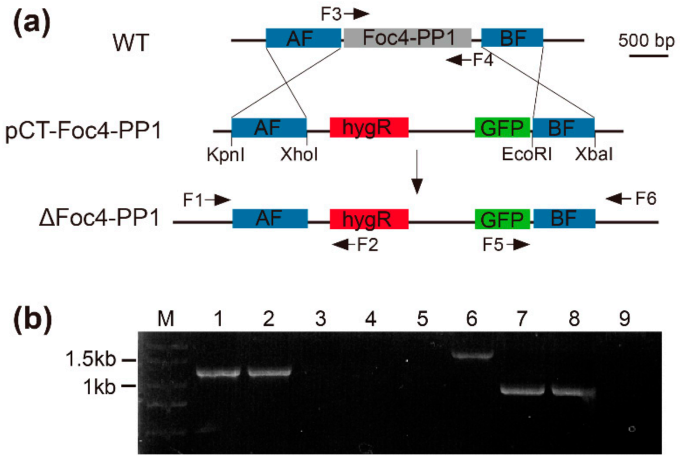

Figure S1. A schematic representation of targeted deletion of the Foc4-PP1 gene. (**a**) The Foc4-PP1 deletion constructs pCT-Foc4-PP1, which contains the hygromycin resistance (hygR) and green fluorescent protein (GFP) cassettes flanked by the upstream (AF) and downstream (BF) segments of the Foc4-PP1 gene, was used for the replacement of the Foc4-PP1 locus using double crossover recombination. (**b**) The identification of the Foc4-PP1 deletion using the primer sets listed in Table S1. Lanes 1, 4, and 7 were amplified by PCR with F1/F2, F3/F4, and F5/F6 primers of ΔFoc4-PP1-12, respectively. Lanes 2, 5, and 8 were amplified by PCR with F1/F2, F3/F4, and F5/F6 primers of ΔFoc4-PP1-13, respectively. Lanes 3, 6, and 9 were amplified by PCR with primers F1/F2, F3/F4, and F5/F6 of Foc TR4-14013 wild-type strains, respectively.
